# New public health approaches to palliative care, a brave new horizon or an impractical ideal? An Integrative literature review with thematic synthesis

**DOI:** 10.1177/26323524211032984

**Published:** 2021-10-06

**Authors:** Joseph M. Sawyer, Paul Higgs, John D.H. Porter, Elizabeth L. Sampson

**Affiliations:** Division of Psychiatry, University College London (UCL), 6th Floor, Wing B, Maple House, 149 Tottenham Court Road, London W1T 7NF, UK; Division of Psychiatry, University College London (UCL), London, UK; London School of Hygiene & Tropical Medicine, London, UK; Division of Psychiatry, University College London (UCL), London, UK

**Keywords:** community, health promotion, palliative care, public health, social capital, social determinants of health

## Abstract

Access to palliative care for marginalized communities is frequently problematized as a major challenge facing palliative care services. The traditional response of asking what services can do for the disadvantaged has been invigorated by a new wave of public health measures that embrace death and dying as social processes and ask, what can be done together with such communities as partners working in palliative care. Such work has generated a significant amount of academic, social and political interests over the last 20 years; however, we are yet to see a consistent and sustained change in approach from providers. We argue that this is due to inherent tensions that arise when modelling death, dying and loss as a unified and shared social process. Unresolved tensions destabilize the theoretical foundations and risk misrepresentation of core philosophies. In this integrative review of 75 articles, we present previously undiscussed areas of contention drawing from a pan-disciplinary field of theoretical and empirical evidence. We conclude that new public health approaches lack a consistent and unified theoretical approach. From philosophical, ontological and existential ideas relating to how different stakeholders conceptualize death, to the processes by which communities are motivated and their constituent members empowered through responsibilized notions of duty and reciprocity, there is little acknowledgement of the complex tensions at hand. Increasing academic and political initiative alone is not enough to progress this movement in a manner that achieves its full potential. Instead, we must pay greater attention to the tensions described. This article aims to work with such tensions to better define the landscape of collective moral responsibility in end-of-life care. We believe that this is crucial if palliative care is to avoid becoming a technical speciality with community and communitization reduced to a mere technical solution to more profound questions.

## Introduction

Through its holistic human response to death, dying and loss, palliative care has revolutionized the way care is performed and provided at the end-of-life. It is now a mainstream component of health care in the United Kingdom and is internationally recognized as a crucial part of integrated, people-centred health services.^1^ Despite this, reports remain from within the health sector of a lack of compassion and dignity at the end-of-life.^2,3^ Palliative care services have also been criticized for their failure to reach ‘marginalised communities’.^4–6^ Concerns relating to access have been amplified by predicted changes in global demographic profiles that are skewed towards old age, multi-morbidity and frailty.^7^ Those people facing death in advanced age have unique care needs,^8^ yet are poorly accommodated for in traditional palliative care models, especially when they have a health condition other than cancer.^9^

The changing context for end-of-life care is set against a backdrop of broader questions relating to what is perceived as the ‘professionalisation’ of end-of-life care. How this notion is juxtaposed alongside the ever-changing weight and value attributed to ideas of neighbourhood, community, work, culture and family traditions is a source of tension. Increasingly, people are searching for and using frameworks from outside of the biomedical domain to work through such tensions and improve end-of-life care.^10^ One such framework exists under an amalgamation of seemingly interchangeable terms, including a ‘new public health approach to palliative care’,^11^ ‘health promoting palliative care’^12^ and ‘compassionate communities’.^13^

The close association between public health, health promotion and palliative care is not immediately obvious. Traditional public health measures of disease prevention and control have been adopted by palliative care since the 1990s helping to improve access to analgesia and integrate services into the mainstream.^14^ While these remain important concerns, a new public health approach focusses on health promotion and empowerment, so as to increase our own personal resources for life and living. The Ottawa Charter was central to this shift and for the first time placed emphasis on re-orienting health services to strengthen community action, develop personal skills and create supportive environments.^15^ Engaging communities in health projects has subsequently been shown to improve health behaviours and consequences^16^ leading Kellehear to outline his ‘health promoting’ or ‘new public health’ approach to palliative care.^12^

This approach recognizes that it is often not health professionals, but rather friends, family and close social networks that provide the majority of care when someone is dying or grieving.^13^ The support that these people require to carry out such tasks is built up through additional networks of people that link together to create what have been termed ‘Compassionate Communities’.^13^ Such communities can be developed to act as a repository of knowledge, experience and human resource that can be accessed and utilized when someone is dying.^17^ Interest in this idea has accelerated at a rate outstripping any form of sustained change or impact.^18^ Some have alluded to the relationship between public health and palliative care being ‘largely symbolic and tactical in nature’ arguing the need for ‘greater theoretical, practical and critical engagement’.^19^ We argue that tensions extend further than this and are as yet poorly acknowledged and accounted for.

The numerous people involved when someone is dying represent an array of differing ontological, philosophical and indeed existential perspectives on death. Attempting to conceptualize death in a way that unifies and mobilizes entire communities is therefore fraught with complications. The lack of such a consistent approach has implications for how we then delineate responsibility. This is of particular importance when delving into the relationship between professional and lay caregiving services. Such ontological and existential factors have to match the processes and outcomes and therefore necessitate considered formulation. This is especially so when considering performing an intervention, such as those associated with Compassionate Communities. Here, issues relating to the often universally accepted notions for positive change, such as empowerment, compassion, social capital and community, can cut both ways when considered at the level of the individual. Such theoretical inconsistencies can permeate through at an operational level and indeed beyond into the wider structures and frameworks at play.

Such issues necessitate good leadership. This in itself is a complex area that brings with it the slippery subject of power, its use and indeed misuse, and how this then comes to bear on the ontological and philosophical foundations of a new public health approach to palliative care. Taking these factors into account, we also challenge the inherent tensions in using traditional academic and medical structures to evaluate the ‘impact’ of such work. We examine the concept of ‘experience’ and how this may be defined and utilized in the process of evaluation. We hope this may prevent the finer complexities of a new public health approach being reduced to overly simplified policy that satisfies the pursuit of immediately viable and effective solutions to proxy measures of what may constitute ‘good care’ or a ‘good death’.

We argue that by embracing unresolved tensions and contradictions, we have the potential to overcome or accommodate them to provide a level of transparency that is essential to building the collective moral responsibility that the movement adheres itself so strongly to. To this extent, the aim of this review is to identify studies explicitly affiliated to Kellehear’s ideas on new public health approaches to palliative care and systematically map the theoretical underpinnings and assumptions upon which they are based. In doing so, we hope to identify and examine some of the inherent tensions with the intention of providing a theoretically sound platform for the development of future work in this field.

## Methods

From this point forward, we use ‘new public health approaches to palliative care’ as an umbrella term to encompass concepts, including health promotion, compassionate communities, compassionate cities and any other measure relating to community engagement and asset-based interventions. In many cases, new public health approaches were found to breach the boundaries of what might be considered to be a systematic scientific approach. Rather, they take the form of a social ‘movement’, suggestive of a much broader socio-political agenda. We therefore refer to new public health approaches as a ‘movement’ throughout this article. We summarize the empirical and theoretical literature, integrating theory from a range of disciplines, so as to provide a more comprehensive understanding of new public health approaches to palliative care. We followed the overall framework put forward by Whittemore and Knafl for rigorous integrative literature reviews,^20^ for data analysis, we used Braun and Clarke’s^21^ flexible and reflexive method for thematic analysis.

### Problem identification stage

We sought a theoretical framework that would allow us to depart from a solely descriptive process and allow us to review the literature through a more analytic lens. This was crucial in addressing the overall research question of what might be considered the constituent components of new public health approach to palliative care, where and what are the underling tensions, and how do such tensions impact on the development of the movement.

Through general reading that followed a brief scoping search of the literature, we understood new public health approaches and compassionate communities in particular, to be socially constructed, complex and unpredictable entities. It was decided that there was a need to understand these concepts in relation to human behaviour in a more interpretivist approach. With the intention of embracing the naturally inherent complexity within such concepts, we also drew on critical realist philosophy when analysing data. Although not explicitly, a ‘realist review’ this framework enabled us to think what might be working and why, and also for whom and in what context thus helping to refine our theoretical arguments.

### Literature search stage

A search strategy was designed and carried out by J.M.S. in March 2020. The databases CINAHL, Embase and Medline were searched using the following terms: Health promotion, public health, social change, new public health, palliative care, end-of-life care, terminal care, incurable, hospice, palliative medicine, community, community engagement, community resource, social networks, caring networks, social capital, asset-based approach, compassionate communities/cities, community development, social support, community participation and community support.

**Figure 1.**
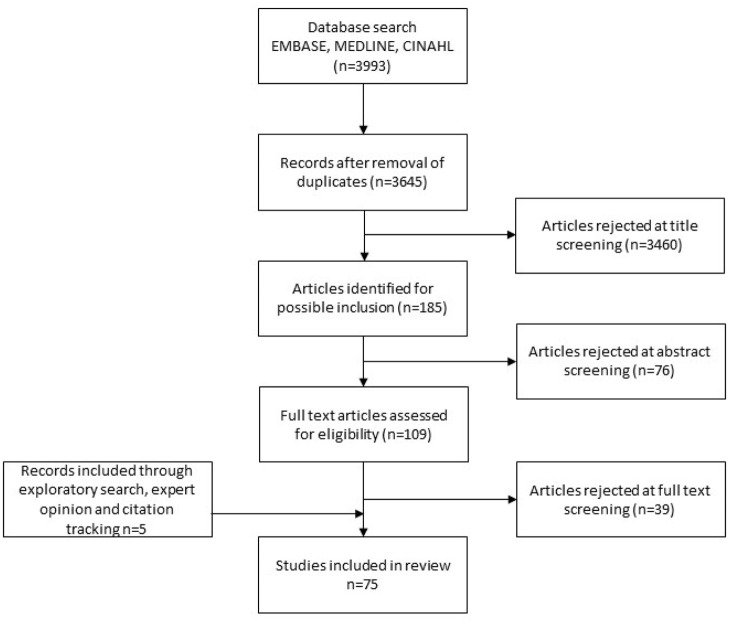
Preferred Reporting Items for Systematic Reviews and Meta-Analyses.

The search included empirical and theoretical studies written in English and either explicitly acknowledged as sitting within the framework new public health approaches or health-promoting palliative care as defined by Dempers and Gott.^22^ Studies describing initiatives or theory relating to salutogenic notions, such as promoting community engagement, nurturing compassionate communities and adopting asset-based interventions to palliative care, were also included. Details of the selection process are outlined in the Preferred Reporting Items for Systematic Reviews and Meta-Analyses (PRISMA) chart^23^ (Figure 1). Details of the individual studies are given in Table 1.

**Table 1.** Literature Chart of Included Articles.

**First author**	**Aim**	**Participant/sample**	**Design/method**	**Key finding**	**Country**
**Allen 2012** ^24^	Examine EOLC pathways in context of health promotion	NA	Theoretical	Biomedicine predominates, ACP as a means of health promotion	The United Kingdom
**Aoun** **2018** ^25^	To determine who provides bereavement support in the community	Bereaved people	Cross-sectional survey	Strengthen compassionate communities to support bereaved people	Australia
**Buckley 2002** ^26^	Understand an educational strategy to encourage health promotion	NA	Theoretical	Holism as a framework for teaching the practice of health-promoting palliative care	The United Kingdom
**Byock 2001** ^27^	Conceptual framework is presented that describes pertinent whole-community characteristics, structures, processes and outcomes	NA	Theoretical	The framework offers a map for whole-community research, intervention and evaluation with the goal of changing the community culture related to life’s end and thereby improving the quality of life for dying people and their families	The United States
**Abel 2013** ^28^	Propose a new model of palliative care	NA	Theoretical	The person with illness is the centre of a network that includes inner and outer networks with communities and service delivery organizations	The United Kingdom
**Abel 2018** ^29^	Stress the importance of networks of people	NA	Theoretical	Communities can offer possibilities for support	The United Kingdom
**Abel 2018** ^29^	To propose a practice model on a public health approach to palliative care	NA	Theoretical	Inadequacy of a solely clinical model of care	The United Kingdom
**Abel 2018** ^29^	To evaluate a population health complex intervention of an enhanced model of primary care and compassionate communities on population health improvement and reduction of emergency admissions to hospital.	People with emergency hospital admissions	Cohort retrospective study	Intervention was associated with reduction in unplanned hospital admissions with an associated reduction in health care costs for the area	The United Kingdom
**Conway 2008** ^30^	Examines an emerging shift to public health approaches in the ‘developing world’ and the ‘developed world’	NA	Theoretical	Community-based approaches can contribute to a much broader agenda and potential for palliative care	The United Kingdom
**Correa 2018** ^31^	Describe an existing model of care in primary care setting	Case study	Case study	Approach can provide more equitable care	Brazil
**De Lima 2016** ^32^	To propose a role for the integration of public health into the community setting	NA	Theoretical	Implementation must be prioritized and planned by the health administrators as a priority public health issue, not only to improve the global efficiency and appropriate use of resources in the system, but also to improve the quality of care for patients with life-limiting illnesses and to relieve suffering	The United States
**Dempers 2017** ^33^	Explore understandings, uptake and nature of public health approach in NZ hospices	Hospice leaders	Mixed methods	Public health approach is a priority, however, there is poor understanding of the principles	New Zealand
**Fook 2010** ^34^	Explore the use of critical reflection in supporting health-promoting palliative care	NA	Case study	Different assumptions exist depending on the stakeholder, critical reflection as a way of resolving this	The United Kingdom
**Gomez-Batiste 2018** ^35^	Describe the aims, activities and phases of a compassionate community project	Volunteers	Mixed methods	Joint leadership with clear aims and targets is important to success	Spain
**Gott 2020** ^4^	To highlight sex inequalities regarding end-of-life caregiving and palliative care and show how these intersect with other social determinants of health	NA	Review	To argue for a paradigm shift in palliative care research, practice and policy to incorporate a focus on sex especially in communities	New Zealand
**Gott 2018** ^10^	Explore the role of community at the end-of-life for people in advanced age	Patients and relatives	Qualitative interviews	Providing community-based care for people of old age is fraught with tensions and needs to be flexible and responsive to the unique needs of those in advanced age	New Zealand
**Green 2015** ^36^	Critical description of the relationship between communities and the public health profession	NA	Theoretical	Community brings both positives and negatives and should not be romanticized. The context in which such programmes are implemented is crucial and a ‘one model solution’ is unlikely to be of use	The United Kingdom
**Hartley 2016** ^37^	To situate hospices within the broader community and describe methods of engagement	Vignettes	Theoretical	Hospices as part of a community and a necessary change in attitude from within mean stream health care professionals	The United Kingdom
**Hazelwood 2018** ^38^	Describe an established public health and palliative care alliance	NA	Theoretical	Enthusiasm exists for the topic, local ownership is crucial for success, there are established barriers, evidence is a complicated issue	The United Kingdom (Scotland)
**Horsfall 2013** ^39^	Illuminate the quality and effect of informal caring networks at the end-of-life	Patients and relatives	Qualitative	Carers successfully mobilized to provide care at the end-of-life. This contributed to social capital and towards the concept of a compassionate community	Australia
**Horsfall 2014** ^40^	To develop an understanding of how formal and informal carers work together when care is being provided in a dying person’s home	Professional and voluntary caregivers	Qualitative	Combinations of formal and informal caring networks are essential to support people and their primary carers. Formal service providers do little to establish, support or maintain the informal network	Australia
**Horsfall 2018** ^41^	To understand if and how network-centred care supports carers of the dying while developing a whole of community approach	Caregivers, service providers, community members	Qualitative interviews	Service systems need re-orienting to place caring networks as central to the caregiving process	Australia
**Huang 2018** ^42^	Describe the five basic principles of the Taipei declaration on health-promoting palliative care	NA	Theoretical	Integrate palliative care into public health policy, create supportive environments, strengthen community actions, develop personal skills, re-orient health services	Taiwan
**Johansson 2012** ^43^	To describe how caregiving can contribute to social capital as opposed to draining it	Community members	Case studies	There must be attention to the way that caring is organized, supported and recognized, which can make the difference between a positive and a negative experience	Sweden and Australia
**Kellehear 2007** ^44^	Critical analysis of health promotion from within the specialities of palliative and bereavement care	NA	Theoretical	Death as a social experience that requires active community-based support and development	The United Kingdom
**Kellehear 2008** ^45^	To summarize the main rationale and concepts of health-promoting palliative care, list some of the key policy and academic writing on the subject and provide one example of its practice	NA	Theoretical	Strengthen a community’s capacity to support to help the individual	Australia
**Kellehear 2009** ^46^	Critical commentary on policy approach to death and dying in dementia	NA	Theoretical	Current policy is overtly clinical and has disproportionate attention to physical and psychological domains	The United Kingdom
**Kellehear 2010** ^47^	Outline of key approaches for health-promoting palliative care	NA	Theoretical	Techniques for building community capacity for the activities of caregiving	The United Kingdom
**Kellehear 2013** ^13^	Examines a health-promoting policy, its conceptual origins and importance to current practice	NA	Theoretical	Health as a shared responsibility	The United Kingdom
**Kellehear 2016** ^48^	To provide an academic and professional context to the ideas of health promotion and palliative care	NA	Theoretical	Understand how an emphasis on public health with affect efforts to provide clinical support at the end-of-life	The United Kingdom
**Kellehear 2020** ^49^	An argument to act on the social determinants of health in the context of palliative care	NA	Theoretical	A social model of health for palliative care, promote civic engagement and partnership, build and normalize cultural literacy	The United Kingdom
**Kelley 2007** ^50^	To conceptualize a rural communities process of developing palliative care	Health care providers	Qualitative	Theoretical model whereby palliative care is developed in sequential phases	Canada
**Kelley 2018** ^51^	Integrate palliative care services into an Indigenous community	Primary carers	Qualitative	Evidence for the implementation of a public health approach to palliative care in an Indigenous context	Canada
**Kumar 2007** ^52^	Discuss the evolution and functioning of the neighbourhood network in palliative care in Kerala, India	NA	Case study	Key successes and learning points from a practical example	India
**Kumar 2013** ^53^	Discus the state of palliative care in India	NA	Mixed methods	Potential for a public health approach to improve access to palliative care	India
**Leonard 2015** ^54^	To analyse the caring networks of people with a terminal illness who are being cared for at home and identifies changes in these caring networks that occurred over the period of caring	Informal caregivers	Mixed methods	Networks increase in size through caregiving, there is importance in the relationship between core and peripheral network members	Australia
**Lewis 2013** ^55^	To summarize the literature on social capital, well-being and quality of life for key outcomes to inform a model of social capital in palliative care	NA	Literature review	Social capital can provide structure for understanding how care is provided at the end-of-life	Australia
**Librada Flores 2018** ^56^	Describe a method for developing compassionate communities	NA	Theoretical	A framework for the development of compassionate communities	Spain
**McLoughlin 2016**57,58	Reflection on the adaption of the world café movement in the context of end-of-life	NA	Theoretical	Preparation, presentation and pilot evaluation of compassionate communities café conversation	Ireland
**Mills 2015** ^59^	To identify and examine community-based activities around death, dying and EOLC which might reflect a health-promoting palliative care philosophy	Local community groups	Qualitative	Potential to enhance health service provision while restoring community agency	Australia
**Mills 2016** ^61^	To discuss future directions for community engagement as a public health approach to palliative care	NA	Theoretical	The use of the arts, social media and appreciative enquiry in contributing to asset-based public health approach	Australia
**Mills 2019** ^62^	To discuss the contribution of palliative nursing to health-promoting palliative care	NA	Theoretical	Valuing compassion expressed within social networks of crucial importance	Australia
**Murray 2012** ^63^	Highlight the need to provide palliative care in the East Mediterranean	NA	Review	Building capacity to benefit access can be done through a public health approach	East Mediterranean
**Murray 2010** ^64^	Highlight the challenges to palliative care	NA	Theoretical	Addresses five key challenges to providing palliative care on a global scale	The United Kingdom (Scotland)
**Murray 2008** ^65^	To discuss the importance of primary care in providing community-based palliative care	NA	Theoretical	Relationship between primary care and specialist palliative care and also the wider community is important, cannot neglect the role of primary care physicians	The United Kingdom (Scotland)
**Murray 2016** ^66^	To describe a renewed vision and shared purpose with respect to EOLC	NA	Theoretical	Clinicians have the potential to support communities to play a role in EOLC	The United Kingdom (Scotland)
**Murray 2015** ^67^	Document barriers and facilitators for palliative care in the community and to provide a resource toolkit for use by professionals to develop services	Professionals	Systematic review and survey	Toolkit can help community-based palliative care services develop	The United Kingdom (Scotland)
**Noonan 2016** ^68^	To explore the concept of death literacy	Relatives and health care providers	Mixed methods	Describes a conceptual framework for understanding the outcomes for a public health approach to palliative care	Australia
**Abel 2018 curriculum** ^29^	To critically evaluate the UK palliative medicine syllabus in relation to contemporary palliative care policy	NA	Theoretical	Current syllabus not accommodating of current policy	The United Kingdom
**Patterson 2014** ^69^	Scottish perspective on health-promoting palliative care	NA	Theoretical	The amount and the diverse nature of health-promoting palliative care activity currently taking place in Scotland indicate that many organizations perceive the importance of this issue and are taking action to address it	The United Kingdom (Scotland)
**Paul 2016** ^70^	Explore the role of hospices in working with schools to promote education and support end-of-life and bereavement	Hospice staff, children and parents and schools	Mixed methods	Innovations identified to help hospices engage with communities	The United Kingdom (Scotland)
**Pereira 2018** ^71^	Implement an education programme and investigate its impact	Teenagers	Mixed methods	Programmes contributed to creating an awareness of palliative care	Portugal
**Pesut 2014** ^72^	An exploration of rural palliative care with a focus on responsibilities that support good care	Community members	Qualitative	The complexity of responsibility and how this is negotiated	Canada
**Prince-Paul 2008** ^73^	To advance understanding of the social well-being domain, a dimension of quality of life, from the perspective of dying individuals	People with cancer	Qualitative	The importance of close relationships at the end-of-life	The United States
**Rosenberg 2010** ^74^	Critical review of the literature relevant to the conceptual foundations of health-promoting palliative care	NA	Review	Health promotion is amenable to the core concerns of palliative care, research needed on impact	Australia
**Rosenberg 2011** ^75^	Critical discussion on the role of responsibility and privacy at the end-of-life	NA	Theoretical	Demonstrates the complex and dynamic nature of the transaction between the dying person, their family and the palliative care service providers	Australia
**Rosenberg 2016** ^76^	Evaluate progression in palliative care	NA	Theoretical	Evidence is required for funding bodies and policymakers, without it, putting the ‘public’ into public health may remain an array of short-term, project-based activities at the edges of clinical care	Australia
**Rosenberg 2018** ^77^	This study examines the ways health systems, services and individual health care professionals influence care at home at the end-of-life	Informal carers	Qualitative	To upscale and maintain the successful efforts of people to care for someone at home at the end-of-life, formal service providers must integrate into caring networks, positioning themselves as integral members of caring network	Australia
**Ross 2018** ^78^	Understand how nursing community may engage with compassionate communities	NA	Theoretical	Compassionate communities must work alongside services and not replace them	The United Kingdom
**Rumbold 2014** ^79^	Understand how bereavement care fits into the model of health-promoting palliative care	NA	Theoretical	Care around grief should be of equal importance to the care of the dying person	Australia
**Sallnow 2014** ^80^	To present a definition and a conceptual model of community engagement for EOLC services and the communities they serve	NA	Theoretical	Community engagement is a spectrum of activity	The United Kingdom
**Sallnow 2016** ^11^	To review the evidence relating to the impact of a new public health approach to EOLC	NA	Review	Evidence exists and the approach can influence complex issues, such as community capacity, well-being and social isolation	The United Kingdom
**Sallnow 2016** ^11^	To consider the research challenges related to examining new public health approaches to EOLC and how learning from more traditional or classic public health research can influence a future research agenda	NA	Theoretical	By bringing together strong traditional methods, such as analysis of longitudinal population-level data with participatory approaches that draw on communities’ experience and aspirations for care, the authors suggest that new and improved opportunities exist to evaluate the impact of participatory approaches	The United Kingdom/Belgium
**Sallnow 2017** ^81^	To understand the impact a new public health approach to EOLC project can have when initiated through a hospice	Caregivers, palliative care professionals, patients	Mixed methods	Collective social capital can be used to understand the role of reciprocity and interdependency between the lay and professional worlds	The United Kingdom
**Sawyer 2018** ^82^	To describe the informal resources and networks available to persons affected by multidrug-resistant tuberculosis, how they are accessed and how they are integrated into everyday lives	Patients and relatives	Qualitative	Community-based approaches may extend human resource and supportive networks	India
**Sawyer 2019** ^83^	To understand how social capital is used in EOLC in dementia	NA	Review	Complex systems, such as communities and social capital, do not offer uniformly positive outcomes	The United Kingdom
**Sinclair 2014** ^84^	To explore perceptions of Noongar community members towards ACP, while developing culturally appropriate information resources for use in community and hospital settings	Community members	Qualitative	Culturally appropriate methods of engaging Aboriginal people in ACP discussions should include Aboriginal health workers and take a whole of community approach to awareness raising	Australia
**Sirianni 2019** ^85^	To explore why health-promoting approach has not been adopted in the Canadian context	NA	Theoretical	Compassionate communities as a potential solution to palliative care policy issues	Canada
**Stajduhar 2010** ^86^	A critical analysis of health promotion and ‘empowerment’ in the context of palliative family caregiving	Family caregivers	Theoretical	In particular, there is a risk that empowerment may be operationalized as transferring technical and medical care tasks to family caregivers at home	Sweden
**Stjernsward 2007** ^14^	To explore public health strategy in palliative care	NA	Theoretical	Framework for integrating palliative care into a country	The United States
**Stjernsward 2007** ^14^	Describe principles of public health national palliative care programmes	NA	Theoretical	Four foundation measures to help establish palliative care	The United States
**Lindqvist 2016** ^87^	To share ideas, experiences and reflections from the early stages of a research programme looking to develop health-promoting palliative care	Community members	Action research	Including publics in public health research, means also including ourselves and making public many of the reflections, the mistakes and the experiences we all have, to foster collective learning	Sweden
**Tompkins 2018** ^88^	To present the historical development of the public health approach to EOLC in Canada, including its successes and hardships	NA	Theoretical	The evolution of the movement has not been difficult. Some confusion within core concepts has led to some downfalls. More work needed and support for key stakeholders is key	Canada
**VanderPloeg 2001** ^89^	Understand health promotion in the context of occupational therapy	NA	Theoretical	Health promotion and palliative care have a lot in common with respect to an occupational perspective	Australia
**Williams 2011** ^90^	To examine how Canada’s Compassionate Care Benefit operates as a public health response in sustaining informal caregivers providing Palliative/End-of-life care	Caregivers	Qualitative	Financial benefit has potential to relieve some of the burden caregivers experience when trying to balance employment and other roles while caring for a patient at the end-of-life. As such, it marks a step in developing a public policy that addresses the public health issue of caregiver burden	Canada

ACP, advance care planning; EOLC, end-of-life care.

### Data evaluation

Due to the challenge of applying uniform quality criteria to a diverse range of research designs, integrative literature reviews do not lend themselves to evaluating the quality of data.^20^ Furthermore, this review focussed on the conceptual frameworks adopted and the underlying theoretical assumptions as opposed to the outcome of research findings per se. As such, quality was less relevant compared with a traditional systematic review looking at more homogeneous data. The studies in this review have therefore not been subject to formal quality assessment.

### Data analysis

The analysis of the data was based on the flexible and reflexive method for thematic analysis proposed and refined by Braun and Clarke.^21,91^ We understood meaning and knowledge to be situated and contextual while researcher subjectivity was conceptualized as a resource for knowledge production as opposed to a source of bias. We used reflexive thematic analysis in a predominantly deductive way, using existing research and theory from the field of sociology and social gerontology relating to communities, social capital, age, ageism and stigma as well as drawing on critical arguments relating to the relationship between lay communities and the state. We then juxtaposed this theory alongside ideas inductively developed from the examined literature. In an attempt to add ontological depth, and better understand causation, we used abductive reasoning as a final step in the generation of themes and the writing of the article.

Data analysis was conducted using QSR International’s NVivo 12 software. First, to aid data familiarization, J.M.S. reviewed seminal papers and textbooks on the topic in an effort to become grounded in the literature and develop a broad base of terms through which key theoretical components could be understood. The data set was then organized into codes. These were based on the key components of the health-promoting palliative care, typically containing at least one observation or facet. We use the term ‘code’ as defined by Braun and Clarke^21^ who conceptualize this as ‘an analytic unit or tool, used by researcher to develop (initial) themes’. Rival theories were highlighted and discussed in memos that were then linked to relevant codes.

Codes and memos were analysed for patterns, content and meaning in relation to the overall research question. Evolving ideas and tensions were discussed in weekly seminars that sought to identify existing pan-disciplinary theoretical frameworks that would corroborate or refute emerging theory. Patterns were then grouped together into sub-themes and then subsequently themes. The idea being to reach a nuanced analysis that provides overarching, multidimensional and ‘meaning rich’ themes. Themes were further refined during the writing process when they were reviewed by J.M.S., P.H., J.D.H.P. and E.L.S. until consensus was reached.

Data analysis was heavily disrupted by the COVID-19 pandemic with the lead researcher (J.M.S.) being redeployed back into full-time clinical work in the National Health Service (NHS). In this role, J.M.S. worked as a community-based palliative care physician. The experiences in working as a palliative care physician through the pandemic and the influence this had on data analysis was discussed within the research team following a return to academic work. There is no doubt such a role has influenced analysis of data, being open to this and embracing it as a resource for knowledge production is noted as a key component to the methodology. It was decided that actively challenging personal experiences through the systematic evaluation of existing empirical and theoretical data including actively seeking out opposing or ‘rival’ theories to the researchers lived experience is recognized as adding integrity and strength to the methods and subsequent findings.

## Results

Of the 75 articles identified, 37 were theoretical and 38 were empirical studies. Articles originated from the United Kingdom, Ireland, Australia, India, New Zealand, Spain, Portugal, Belgium, the United States, Canada, Taiwan, Sweden, Brazil and the East Mediterranean. Themes are divided into four sections: philosophical perspectives, processes, structures and finally experiences. The philosophical underpinnings of the movement are evaluated first to understand how death is constructed as an idea thus providing a lens through which the following sections can be interpreted.

## Philosophical perspectives

Historically, the way we conceptualize death directly corresponds to the resources developed to support it. For example, the power of religious social imagery concerning death developed a corresponding social role for those people experiencing it.^44^ Similarly, where death is characterized by the mere absence of life, the roles of those experiencing death are hidden and lack legitimacy.^92^ Understanding death from the perspective of its physical processes is recognized as reinforcing the notion of death as a final end point simultaneously shifting the balance in power from the dying person to the doctor.^93^ The articulation of palliative care, public health and health promotion in closer terms has resulted in a new, more expansive understanding of death and the experiences it brings. The key components, as distilled from the literature, are outlined below.

### The whole as greater than the sum of its parts: death, grief, caregiving and bereavement as one totality

Death is conceptualized as a process that both shares and requires an interdependence and social connection with processes of grief, caregiving and bereavement.^44,39,94,51–79^ New public health approaches reject the notion of palliative care and bereavement as separate entities and construct a paradigm that embraces the totality of end-of-life experiences placing them together as shared social experiences.^44,39,51,79–95^ This is reflected in the development of compassionate communities that provide a social framework of support for problems that exist in the social world.^13^ In doing so, the movement subtly acknowledges the notion that there are unseen and unquantified mechanisms at play that become lost when the whole is unpacked into its constituent components.

### A shared breath, a shared life and a shared death

A dominant theme from within the literature was that of death as a shared social *process*.^44,39,51,79,25–98^ Authors draw upon the idea of the universality of death, grief and loss and a shared impermanence held between all living things.^44,59^ Death therefore is not characterized simply by the absence of life, beyond which there is social vacuum. Rather, it is argued that death can be ‘full of life’ through the interpersonal experiences that the process of dying, caring and bereavement brings.^44^

Consequently, death is understood as having its own life course where the associated transitions and transformations unfold over time to generate social change. In this way, death as a process has its own legacy which can ‘increase compassion, empathy and social sensitivity’ while the dead play important roles for the living as role models and motivators of change.^44^ Such a notion is encapsulated in the sharing of breath; that the breath we take was someone else’s moments ago and will become part of someone else soon after we let it go. It continues to exist through others long after it has passed through us.

Framing death in such a fashion raises important questions relating to responsibility, duty and motivations in relation to end-of-life care. The multiple stakeholders involved in providing end-of-life care, families, communities, workplaces, professional services and the state, do not share a unified philosophical approach to death thus creating a discordant and fragmented response.

### Dancing your own dance: negotiated responsibility and the moral landscape

Morality has been described as being born out of shared understandings of ‘assigning, accepting and deflecting responsibility’.^99^ Where responsibilities are not clearly understood or felt, fragmented care, power struggles, service gaps and moral distress can prevail.^72^ It follows that for the human interdependencies to be realized as actual connections rather than theoretically constructed ideals, there is a necessary negotiation of responsibility that follows. The literature talks of compassion as a ‘moral imperative’ upon which the movement is built.^44^ There is also mention of how ‘shared responsibility’ is a ‘hallmark’ of compassionate communities.^25,30,33,61^ However, there is less clarity on the negotiation of responsibility and how this then creates a compassionate moral landscape.

In the context of care, such negotiations, and their related outcomes, depend significantly on the cultural context and a society’s position on the spectrum between collectivism and individualism.^100^ Central to individualism is the autonomous individual who exists in a largely heterogeneous and competitive society. The breadth of variety promotes a tolerance of difference yet breeds dependence on services. The solidarity or interdependence here is rational, for example, I choose which service to use and how much to pay for it, but recognize I am also interdependent on its function. Collectivism is founded on the interdependent self^101^ and exists largely in homogeneous cultures. The norms, roles, rules and values of society are well established and promote a degree of duty yet are difficult to break free from.^100^ In reality any given culture incorporates a mixture of both, for example, families tend to adhere to collectivist principles, while the market is inextricably linked to individualist social relationships.^100^

At a surface level, new public health approaches would seem to endorse a reconstructive collectivization, that is, the redesigning of our individual lives in an attempt to collectively confront death through our own autonomous individual lives. In this way, responsibility is governed by a form of duty and maintained through social norms. It follows that such a model risks ostracizing those who disagree or are unable to partake in such activities. Where such people are free to leave without repercussion the collective culture is at risk of a gradual erosion.

At a deeper, more abstract level, the idea of a shared responsibility is suggestive of an innate connection held between all living things that breeds a fluency to the roles we play in life and death. Here, responsibility is less of a cognitive process and more something we feel which compels us to move and act in a way that is unique to us as individuals. This notion gave rise to the theme of ‘dancing your own dance’ which evolved in response to the dilemma of negotiating responsibilities.

To understand this notion better, we must combine knowledge relating to collectivist and individualist philosophies with more creative ideas on how responsibility is given the space and time to evolve and be felt within us. Part of this process involves understanding where professional services sit within the paradigm of death as a ‘shared social process’.

### Creating, holding and nurturing space: the intersection between public, professional and lay services and the implications on responsibility and duty of care

New public health approaches to palliative care incorporate ideas on health promotion as a means of disease prevention and harm reduction. Such principles demand greater public responsibility, yet they also create bureaucratized systems that demand compliance rather than nurture shared responsibility. In this section, we discuss these tensions in the hope of finding a clearer path forward.

The literature describes the idea of a compassionate city.^49,29^ At one level, a compassionate city is understood to hold some degree of responsibility for end-of-life care. This is clearly not at an individual level, but perhaps more in creating an accepted space for citizens to perform some of the ‘work’ of caring.^41^ Organizations are then able to respond to local needs rather than vice versa leading in theory, to more equitable care.^102^

Within this concept, and interdependent upon it, is nestled the idea of a compassionate community. Such communities are described as having an ‘outer network’ of communal support that lies in relation to an ‘inner network’ of private domestic relationships.^10,17,39^ In its broadest sense, a compassionate community is conceptualized as sharing some responsibility for end-of-life care, yet the specifics of what it is responsible for and how this is held between networks are unclear.

At the level of the family or an inner circle of care, the culturally embedded norms of kinship present duty as a concept that is distinct from responsibility and more in line with some form of obligation.^103^ Parent–child relations are in some cases founded on norms of obligation, how these norms operate and are created within a compassionate community is relatively unexplored yet crucially important. While there are perhaps clear principles which can be referred to when considering the ‘proper’ thing to do, there is no consensus regarding what is reasonable to expect.

At the level of the patient and the professional, there is a more defined, although not binary locus of responsibility. The difficulty arises when responsibility is viewed at a population level. First, professional services do not have the capacity to manage such responsibility, and second, they are considered by some to hold a benign, yet enduring paternalism that is a philosophical and structural barrier to the paradigmatic shift of conceptualizing death as a shared social process.^43^ Framing the problem in public health terms is a logical step to fostering the human resource communities’ offer and building political capital, but conceptually, the locus of responsibility remains rooted in the professional domain. This represents an enduring tension across the literature and a significant barrier to the progression of the movement.

Interdependent on any form of responsibility lies the question as to what motivates a person or group of people to act or assume accountability, both in the first instance and then in a sustained fashion. If compassionate communities are motivated purely by altruism, driven by ideas of interdependence and compassion as a moral imperative, they will be subject to the previously described limitations of a collective culture. Furthermore, it cannot be assumed that all people in all communities are altruistic. While altruism can be fostered via the responsibilities held at a civic or organizational level, the reality is that while communities bring companionship, practical support and comfort, they also bring ignorance, rejection and patronization.^43^ Furthermore, altruism is difficult to implement systematically and can quickly lead to bias.^104^

If motivation is modelled on social exchange theory^105^ and reciprocity, that is, that a compassionate act is exchanged for another or some other form of ‘capital’, there are still inherent tensions. For example, many people at the end-of-life are unable to reciprocate acts of care thus giving rise to the need for professional services. In such an approach, care may be ‘commodified’ and subject to market rules. This is in contradiction to understanding death as a ‘shared social process’. There is some evidence that under the paradigm of shared social responsibility, reciprocity is not defined in terms of simple dyadic transactions.^81,106^ For example, where an act of care or compassion is unreciprocated due to ill health of the recipient, then the benefactor will seek reward elsewhere in the community.^83^ Where these pathways are cultivated, this can, in theory, lead to the building of community capacity. There is therefore a fluidity to the idea of responsibility and the sharing of roles may need to be more commonplace. Traditionally described in the biomedical literature as a source of burden and distress,^86^ the sharing of roles can also be perceived as a fulfilling and rewarding process.^40^

The fluidity regarding responsibility and motivation described within the literature gave rise to the overall theme of ‘Creating, holding and nurturing space’. Under this paradigm, compassionate communities unify professional, public and lay services by forging a space that permits a fluidity to responsibility that then allows people to find their own personal way at the outset while giving room for others to move in and support them at times of difficulty.

### Processes over outcomes, lenses as opposed to interventions

The idea that how the end-of-life unfolds is of greater importance than any single measurable outcome is central to new public health approaches.^30,68,46,38,45,50^ Using the example of responsibility and duty; while a lay person may accept a caring responsibility, it does not necessarily mean that the experience is perceived in net positive terms and will contribute to community capacity. It is the process through which responsibility is negotiated and the moral landscape that is constructed that ultimately decides this.

That said, there is a tendency in the literature to use a new public health approaches to demonstrate effect on well-established outcomes, such as preferred place of death and avoiding hospital admission.^107^ This is reflective of the more biomedical palliative care literature that speaks in terms of outcomes that may be interpreted as ‘positive’ or ‘negative’. For example, a home death may be perceived as ‘good’ or a transition in care from home to hospital or care home perceived as ‘bad’. This infers a more traditional public health perspective and reveals the tension between defining death as a shared social process with varied outcomes as compared with a clinical process with defined end points amenable to focussed interventions.

It follows that new public health approaches have somewhat of a fraught relationship with professionally instigated interventions. On one hand, intervention to aid community development is a central facet to the movement.^13,49,102^ While such initiatives are developed in response to the needs as identified by members of the community^13,17,102^ and seek solutions via collective community resource rather than upscaling private or government-based professional services,^102^ they nonetheless have the potential to demand conformity and narrow the locus of responsibility.

On the other hand, if death is viewed as a shared social process, new knowledge, skill and experiences evolve naturally as a direct response to the challenges of caregiving through the dying process. What is important is how these grow organically and ‘ripple out’ into the world, contributing to a greater body of collective resource. Initiatives are therefore not required to make the ripple, but instead give it the momentum it needs to aid community development. Without an appreciation of such processes, interventions have the potential to wield a power that generate entrenched social norms and demand conformity. Viewed in such terms, it is less an *intervention* that is required but more a platform or stage that affords the space for networks to generate and multiply. Where a change is required, it may be as subtle as creating a lens which offers a different perspective and set of solutions.^72^

### Permitting both joy and grey hollows: the meaning of suffering in relation to the idea of a ‘good death’

Ultimately, the fundamental purpose of new public health approaches is to enhance feelings of support and security at a time of immense sadness and vulnerability. To achieve this, there needs to be some form of understanding as to how we view human suffering and what should be done in response to it.

The specialty of palliative medicine, and the medical world in general, has been influential in promoting the idea of a ‘good death’.^108^ The message is that the pain of death can be managed and supported, and the growing expectation from the public is for people to be comfortable and ‘at peace’ at the end-of-life.^109^ By virtue of logic, the idea of a ‘good death’ immediately implies that of a ‘bad’ death, and within it, elements that are to be remedied or avoided. While individuals and their families may differ in their preferences as well as their normative beliefs regarding what constitutes ‘good’ or ‘bad’, the public message is clear; that there are co-morbidities and symptoms to dying that can be fixed, prevented or alleviated and where this does not happen it somehow translates to a failure in care. The idea of anything other than a ‘good death’ has come to represent something of a failure for palliative medicine. This only serves to distance the speciality from the social roots of death and dying and edge it more closely towards a biomedical model.

A more social approach to suffering is perhaps grounded in the idea that death, much like the life lived until that point, is a ‘mishmash’ of joy and grey hollows.^49,68,43,72,40^ Easing the pain as a loved one dies is an insurmountable task. Nor is it necessarily a virtuous one when creating an environment of complete emotionally sterility would be the only such way of achieving it. While the pain, fear and terrible sadness are there for all to see, there is also companionship, courage, strength, love and compassion in such an example. Caring at the end-of-life must therefore accept, and then accommodate, both the joy and grey hollows it brings.

Rather than something that can simply be alleviated or eliminated, the literature is suggestive of suffering as something with meaning and importance that can be instrumental in creating new social legacies, building social relationships and building a source of new knowledge and hope.^49^ New public health approaches to end-of-life care therefore embrace and work with the suffering that comes with death. By encouraging people to hold it and work with it, so it can be experienced in a healthy way that allows people and communities to transition through and develop from the experience it brings.^49,40^ Conceptualizing suffering in this way is vital to incorporating the full spectrum of community resource into the response to death and dying.

## Processes

We now aim to evaluate the processes at play within new public health approaches to palliative care, using the core philosophical underpinnings as a lens through which to understand them. We encourage the reader to regularly reflect back on the underlying philosophies described to make sense of the forthcoming themes.

### Invoking and awakening the idea of community as an independent agent of care

This theme is an attempt to bring some consensus to the medley of processes mentioned across the literature. Within it, we describe some of the commonly encountered ideas, including empowerment, network building and social capital, and how they align with the philosophical principles described. We identify and discuss some of the inherent tensions by drawing on existing literature from other disciplines before describing our ideas on ‘invoking and awakening community’ as an overall theme.

#### Empowerment

Empowerment is an established concept in new public health-promoting strategies^110^ and is often used as a way of describing shared responsibility.^39,33,38,26,80,36,52^ The idea stems from the notion that humans have a specific set of resources that allow us to adapt to illness and live with the anguish this may bring.^111^

There are various levels of empowerment mentioned in the literature.^80^ At the level of the dying person, it can be seen as a tool to making a healthy adaptation in the face of one’s own mortality, this helps to maintain a sense of control and agency for the person. At the level of community, empowerment is taken to mean the pooling of a community’s collective resource to take action and generate solutions to common problems.^39,52^ At the level of policy and public infrastructure, it is about building a facilitative, non-prescriptive structures that foster such processes.^30^

That empowerment tends to be universally accepted as a force for good within the field of community-based care has implications for family and caregivers, especially at the end-of-life. For example, where people are ‘empowered’ to die at home, family members may be forced to relinquish traditional family roles^86^ while the highly gendered dimensions of caregiving are accentuated.^4,90^ Furthermore, empowering people to work in a more holistic and supportive manner neglects the fact that not all people have the same thresholds to provide such care. People unwilling or unable to work in this way may then be subject to discrimination.^90^

These are examples as to how tensions can arise depending on the level at which empowerment is interpreted. In many ways, empowerment therefore is not something that is bestowed or gifted upon a community but something that is invoked within it. This way the power is allowed to be built collectively, so it is less easily stolen, displaced or corrupted.

#### Network building

Network building is one of the predominant processes in engaging with and empowering communities^40^; however, a range of techniques are described. Authors describe using activism and ‘disruptive innovation’ as a means to achieving the scale and momentum necessary to bring change.^53,64^ Although not formally evaluated in academic terms, the development of such initiatives has the potential to exert significant pressure on the relationships and actions of local and national governing bodies. This influence is important in instigating a cultural shift that brings lasting change. However, the influence of state developed interventions can risk ‘crowding out’ organically developed interventions, thus jeopardizing solidarity within the community.^53^ We could not find empirical evidence for this phenomena in the context of compassionate communities, however, the topic has been discussed at length in the field of sociology and social gerontology in relation to the welfare state.^112^ Opposing this argument is the idea that state interventions draw people into action.^113^ This positively links the growth of community support to the level of giving and receiving.^77^

Community-based interventions can also be founded on existing networks and social assets. Such networks may not develop in a uniform or predictable pattern^41^ as they respond to the complex interplay between love, intimacy, trust and duty. The fields of academia and biomedicine are fundamentally restricted in their abilities to understand how networks move and reorganize at the end-of-life. To help overcome this, there is a need to embrace the capacity held by the arts to provide a creative medium that dynamically engages communities to awaken the spirit within human relationships.^61^ Only then, we can truly foster the full potential within such complexity.

Another approach is to target the structures that impose a blockade on organically developed social networks. This allows for the ‘rippling out’ of knowledge and experience thus allowing the caring capacity of a community to develop naturally. Advocates of such an approach theorize that lay networks of people want to be involved, but lack capacity due to other responsibilities or structures that impede them.^41,40^ At best, professional services and the stream of bureaucratic interventions designed to help do fill the void, at worst they are further structures that obstruct people’s desires to partake in the caregiving process.^40^

#### Social capital

Regardless of the approach to network building, new public health approaches are inextricably linked to the multifaceted concept of social capital. Generally, social capital is understood in three levels: bonding, describing strong homogeneous relationships that foster trust and maintain resource; bridging, between people across difference that generates access to resource and linking, across explicit power differentials allowing for the generation of increased resource.^114^ The effects, however, are not universally positive.^115,116^ At the level of individually bonded ties, there may be damaging close relationships, caring roles may be subject to sex, cultural and economic bias, while the strength of individual ties may bar outsiders thus limiting development. At a community level, disparate pathways to bridged social capital on the basis of social class, race, education and sex can create negative group norms and cause social exclusion.^116^ Macro-level influences through linked social capital can also lead to the possibility of corruption and nepotism.

Framing the concept in palliative care terms, caregiving at the end-of-life is thought to have potential in contributing to social capital through the social interactions it brings.^40^ Caregiving can also help build trust, reciprocity and adherence to a new set of social norms that facilitate social agency.^43^ This is in opposition to the isolation and burden more commonly attributed to caregiving at the end-of-life, especially where old age and dementia are concerned.

However, framing caregiving in such terms neglects the described, more dispassionate, body of work on social capital. Such work would suggest the shaded support of someone far removed from the business of family politics, who can provide a comfort and confidence that is unique in a community that knows everyone’s business, can in itself be a sanctuary.^72^ Attention must also be paid to the levels at which social capital operates. For example, where informal carers are recruited to provide bridged and bonded capital, they may be subject to the all-consuming nature of care^90^ and subsequent economic hardship,^90^ if they are not supported by linked capital that affords them the space to fulfil such duties.

That enhanced social capital is desirable on the part of the dying person is another source of tension. While death may well be universally and routinely experienced, it remains a highly intimate and private matter.^43^ Balancing both the personal and social nature of dying is not straightforward. This is illustrated by work showing people in advanced age can actively withdraw from or resist community support.^10,117^

The capacity of social capital to be operationalized by new public health approaches is therefore very much dependent on a greater theoretical understanding of the tensions at hand. Additional processes and supportive structures are necessary to develop a robust understanding and subsequent plan for integrating informal networks into caregiving. Regardless of the specific approach taken, a consistent undercurrent to the processes involved is to frame death as shared social process by giving precedence to a metaphorical web of ‘betweenness’. Consistently across all studies, death and dying directly breeds new social relationships and experiences, in many ways, therefore, community is not something that is produced but rather invoked and awakened. In this idea of community, identities of awakened and realized individuals challenge and complement each other allowing individuality and originality to enrich both the self and others.

### Embracing that which emerges from the space: the construction, interpretation and use of knowledge

Complimentary to a thoughtful approach to social capital is an understanding of the process by which knowledge is constructed, interpreted and used in the context of end-of-life care. Just as medical professionals have expertise and knowledge relating to disease and clinical assessment, communities hold knowledge relating to customs, values and practices,^34^ while families and close networks hold knowledge relating to a dying person’s identity.^83^ New public health approaches would view all this as knowledge that is relevant to the end-of-life process. This is representative of a paradigm shift from traditional approaches that are based on a hierarchy of knowledge. Where knowledge and expertise are seen to emerge more broadly, people will seek it from the widest range of possible sources. For professionals to actively partake in the exchange of relevant knowledge they must first recognize the importance of socially and culturally constructed knowledge relating to the dying person.^83^ Removing preconceived ideas regarding the importance of professional knowledge can then allow for mutual experiential learning that fosters a cultural literacy drawn from experience.^49^

### Communication flow via ripple effect

‘Social networks lay the foundation for social interaction, which in turn brings about trust, expectations of norms emphasising reciprocity, and cooperation’.^118^ In order for this to happen and for knowledge to be disseminated and learned from, communication flow is of vital importance.^40^ Conversation allows for the social diffusion of insights and experience that have developed in response to the act of dying.^118^ This generates access to knowledge and a repository within which it can be stored and accessed again. Such processes also give rise to a universal language that endorses death and dying as a shared social process. This is described within the literature as the concept of ‘death literacy’.^94,68,40^

The dissemination of knowledge and experience in this way helps produce structural and communal change that embed the work of caring within the community and maintain new public health approaches in line with its philosophical underpinnings.^98^ In these situations, knowledge is conveyed in the form of narrative as opposed to didactic educational techniques employed by professional services. Story telling is thought to create very broad and inclusive opportunities for participation and reflection.^38^ It also reinforces the notion of experiential learning, one of the processes central to the functioning of a compassionate community.

A ripple effect was a term used in the literature and is apt due to the fragility it implies. Taken outside of the social context, this form of knowledge is rarely endorsed and where it is, it is perceived as unquantified and unvalidated. Professional services may also be seen to obstruct the ripples that create such knowledge capacity, this was acknowledged, although not necessarily explicitly, across the reviewed literature.^79,41,72,55,76^

### Dynamic modelling

Crucial to the processes mentioned thus far is the ability of the movement to continually adapt to the changing physical, social and cultural environments.^96^ Processes must recognize and accommodate both slow burning cultural change and also mirror significant shifts in cultural practice that follows major public events. This is a notable distinction from the ‘cultural syndrome’ of collectivism outlined previously.^100^ A health-promoting approach to palliative care requires an adaptive culture that has the ability to transcend challenges and create now patterns of behaviour yet is not limited by professional boundaries.^13,97,89^ Effective partnerships at the individual, community and service levels are required with ongoing consultations with members of the public described as a defining feature.^34^ Without such measures, any cultural change runs the risk of replacing one rigid cultural norm with another. The constant swaying between cultural norms is more akin to some kind of temporary policy fashion rather than a progressive extension of the idea of death being a fundamental part of our human fabric.

## Structures

### Leadership, development and the spectrum of power

Both compassionate cities and compassionate communities require leadership to socially market the concept and enlist the cooperation and creativity of the wider community and potential institutional participants. Leadership must also deliver sustained growth and development of the movement that allows for dynamic modelling. Leadership does not have to come from within the field of health and social care. Indeed, leadership from outside this field would help ground the movement within the philosophy of death as a shared social process. Given the range of enthusiasm for the movement from a variety of sources, this feels like a realistic possibility.^38^ The danger, however, is that communities become ‘responsibilised’ for circumstances that are not of their own making and are beyond their means to effectively address.^30^ There is mention of a locus of leadership being assumed at the hands of the dying person^10,40^ although this concept is complicated by the nature of disease, for example, dementia and the restrictions experienced during very old age. In contrast, professional services hold significant power in addition to their expertise and are uniquely placed to hold such a leadership role. The irony being that the very services possessing the expertise to lead this shift in control are the same services that would be required to yield that control.^75^ There is also a concern that the curriculum for UK health professionals involved in providing such care is of a narrow focus and fails to accurately articulate the foundations of health promotion at the end-of-life.^119^ Given this, there is ample opportunity for the corruption of such power.^48^ Leadership must recognize that a social approach to death cannot be ‘delivered’, but requires careful cultivation and awakening though collaborative engagement. Perhaps most importantly, there is a requirement for leadership to utilize the power and control afforded by the role while ceding such power to allow for organic community development to flourish.

### Spaces

A social model of death is a settings-based approach.^49^ Space is used as a platform for social connectedness that affords room for people to gather and their stories to be shared. Much of the literature is devoted to the idea of home-based care and a home death.^11,68,75,52,53^ The home provides a degree of independence, control and autonomy from professional structures ^68,75,52^ while providing the opportunity for the coming together of intergenerational networks.^68,40^ However, for others, the home can be a place of abuse, exclusion and isolation.^41^ The involvement of professionals at the end-of-life and the ‘paraphernalia of care’ creates somewhat of a hybrid space where there is encroachment of professionalized norms on the traditional culture of the home. A compassionate space offers necessary protection and support yet is reflexive to the identity and agency of the individual at its centre.^17^ Compassionate spaces incorporate and build ‘cultural literacy’ drawn from experience garnered through the valuing of local knowledge.^49^ A compassionate space can be anywhere; in an institution, in the community, or in someone’s own home. It is created by careful and considerate negotiation of responsibility and an adherence and grounding within the philosophy of death as a shared social process. This is an example of how the process by which a space is physically and philosophically constructed is of more importance than the actual space itself and returns us to the underlying philosophies of the movement.

Beyond the home, other social platforms were understood to provide structure to the processes of rippling out and communication flow. The Café Conversations’ activities described by McLoughlin and colleagues^57,58^ and the use of arts and social media described by Mills and colleagues^60^ are examples of how social spaces can be built whereas places of worship, cinemas, parks and even hospitals contain existing spaces that can be recreated in such a fashion when viewed through the appropriate lens.^82,27^

## Experiences

The aim of new public health approaches is to positively influence what is experienced at the end-of-life. Across the literature, experiences tend to be viewed as a product, something that we have some form of control, possession or ownership of. This is in contrast to an experience being something that is played out through sequences of complex drama, something that may surprise us, capture our imagination or thrust us into an arena in which the individual is in contact with something far greater.^120^ Conceptualizing an experience using the latter framework is a useful way to understand the potential outcomes of new public health approaches.

### Self-perpetuating community development and sustainability

There is a notion that good end-of-life care not only requires community development, but also assists it.^28^ This is modelled by the idea that reciprocal actions of compassion are not limited to dyadic relationships.^81,83^ Instead the skills, knowledge and experience developed by caregiving can be utilized in the wider community creating a network for the exchange of such commodities that contribute to an economy of care and compassion.^40,54^ Such notions are also thought to build a community’s capacity to be compassionate and self-sustaining^41,45[Bibr bibr62-26323524211032984]^ while allowing for emergent, community-based leadership that is culturally, economically and needs based. This is, however, far from a universally experienced phenomena and is likely to show significant variation among different age groups and disease types.^10^ The challenge is understanding in what context this phenomenon can be usefully developed.

In order for communities to develop around end-of-life caregiving, it is important that the experience of community growth is not viewed solely as a product with corresponding notions of ownership. Rather the experience of community development may be seen as participation in something more ultimate than one’s own needs or ego. This may refer to notions relating to the ‘spirit’ of a community. Typically, outside of the technical language of academia and palliative care, invoking and awakening the spirit of community is inextricably linked to the work conducted by new public health approaches to palliative care yet conspicuous only by its absence from the literature.

### Continuity of care

Following on from this concept is the idea that informal caregiving networks also provide a continuity of relationship that allows for sustained support in addition to knowledge development.^94^ At a surface level, such continuity bridges the gaps that occur between medical sub-specialties.^17,25,34,93^ This is increasingly vital as the timescale for the dying process in conditions, such as dementia and other chronic diseases are likely to be measured in years. At a deeper level, the experiences, revelations or epiphanies that arise from forming sustained and continuous caring relationships contribute to the notion of death as a shared social process, giving a connection to a metaphorical ‘web of belonging’ that can sustain and nourish even during the darkest of times.

### Shared ownership and awareness of death

Currently health systems hold power that greatly influences the relationships formed.^68^ By sharing the locus of ownership and responsibility and creating a platform for discussion that highlights an accurate reality of the caregiving landscape, people can begin to redress the power balance.^68^ Significantly, some of the greatest challenges faced by caregivers at the end-of-life originate from formalized medical structures and the bureaucratic processes that support them.^41,77^ Furthermore, regulatory systems focussing on risk management, privacy and confidentiality constrain service providers from working directly with informal networks.^41,76^ Such measures have led to some describing the current situation as ‘working together – apart’.^39^ New public health approaches have the potential to provide a universal set of practice principles that allow for shared ownership and responsibility thus overcoming such barriers.

## Discussion

### Summary of findings

This integrative review critically evaluates the underlying philosophical assumptions, processes, structures and outcomes inherent within new public health approaches to palliative care. By integrating rival theories from a range of disciplines, we have highlighted previously unconsidered tensions embedded within the movement. The review attempts to dispassionately acknowledge and work with these tensions to provide a sounds basis for the sustained implementation of a new public health approach to palliative care. Much of this lies in bringing into sharper focus the underlying philosophical principles upon which this movement is founded. Articulating how death and suffering are conceptualized allows us to understand issues of responsibility and motivation in greater detail. Such factors are crucial when looking to enlist the support of a range of stakeholders that hold differing perspectives.

Despite a growing evidence base, which has expanded since our original search,^121–123^, the philosophical underpinnings and their inherent tensions are often poorly discussed. Ignoring such topics runs the risk of a new public health approach morphing into a one size fits all solution that ceremoniously hands back care of the dying to communities as a means of addressing the challenges faced by end-of-life care. To avoid such an eventuality, we suggest that there is a greater need for cross-disciplinary collaboration to help bring such tensions to light. Our attempts to integrate work from the fields of sociology and social gerontology are a step forwards, however, for death to be truly understood as a social process, work from the fields of arts and culture, theology, economics, architecture and design, social work and education must be integrated into research and practice. This will help create a more collaborate culture that can accommodate notions of shared responsibility, knowledge and trust. Furthermore, we encourage academics and activists alike to articulate their underlying philosophical principles in relation to a new public health approach. If the social nature of death is not acknowledged in explicit terms, there is ample opportunity for the development of interventions under the banner of a new public health approaches that only serve to distance palliative medicine from the social roots of death and dying while creating structures that obstruct people in their desire to partake in the caregiving process. Since our initial search and the writing of this article, there have been additional publications in the field that are beginning to address some of these issues^122^; however, as new interventions are developed and implemented,^121^ and the scope is broadened,^123^ there remains an inherent need to acknowledge the arguments laid out in this article.

**Figure 2.**
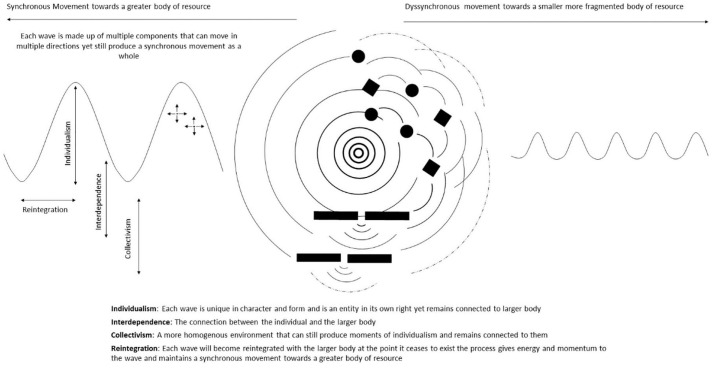
Diagrammatic Representation of Core Themes.

A diagrammatic summary of our main findings can be found in Figure 2. Here, the epicentre of the diagram may represent any singular event with the repercussions represented by the rippling out of waves from this point. Where repercussions lead to the meeting of new bodies or structures, the wave is altered in direction and form and its energy dissipates quickly. Such changes may be welcome or necessary yet produce a wave form that is fragmented with a smaller body of resource as its product.

Where the repercussions are unheeded, a larger wave form exists. In this larger wave form, it is possible to see how notions of individualism, interdependence and collectivism can co-exist and work together rather than be seen as mutually exclusive ideas. As the wave falls, there is a process of re-integration into a larger body that then gives energy and momentum to the synchronous movement of the wave. Framing this in the context of new public health approaches enables us to see how seemingly apparent tensions and contradiction can actually work in unison. However, only by understanding where such tensions lie can new bodies or structures be strategically positioned to create an intentioned or augmenting effect. Similarly, by limiting the addition of new structures, a greater body of resource may be allowed to develop which can have a wider reach and impact. Momentum is built within the wave itself, and crucially, the wave can still hold the seemingly opposing or random movement of individual particles that contribute to the wave form without influencing momentum.

### Strengths and limitations

The use of a broad search strategy has helped build pan-disciplinary links that have generated previously unconsidered tensions within the movement. Naturally, our findings are limited by the breadth of available literature and the human and technical errors inherent within the online search process. While we have attempted to include all relevant articles, it is possible that the search process failed to pick up some articles. We are grateful to the reviewers for highlighting one such article.^124^ Public health and palliative care are most frequently examined from a single professional perspective and the associated blind spots will undoubtedly have led to the dismissal of subsections of community life. New public health approaches to palliative care were felt to be more representative of a movement with a broader socio-political agenda than that of a systematic scientific approach. When considered from this perspective, it can be argued that the data presented are inherently limited by the systematic scientific approach employed in the methodology. This irony is not unnoticed and is taken as another example of the tensions that arise when framing the nature of this work in academic terms. This, however, is a discovery in itself and the systematic nature of the article reinforces a key finding that academic platforms in their current form are fundamentally restricted in their ability to embrace and understand such broad-based concepts within their full complexity. We also recognize that what may be considered a limitation by some is also perceived as strength by others, as such by employing both systematic and reflexive methods to data collection and analysis, we hope our findings can be generalizable to a range of contexts. We have not tested our theories within a lay population, this combined with gaps in the empirical and theoretical data mean that our work will undoubtedly need to be modified as the knowledge base expands.

## Conclusion

We believe new public health approaches to palliative care, including compassionate communities are indeed a ‘brave new horizon’ and hold great potential, not just in revolutionizing the way people view, access and experience end-of-life care, but also in leading the field of medicine and health care into a new ‘moral’ era. Here, co-production and co-design through collaborative practice pave the way for us to recognize people’s voices in terms of what matters to them, establish what is truly important to measure and perhaps bring the notion of healing and ‘whole person’ care to the forefront of medicine and social care.

There is perhaps no better driving force behind such ideas than the way we conceptualize death and dying. Where death is understood from the perspective of one of its constituent components the response can feel constrained, fragmented and muddled. Where death is understood from the perspective of the whole, the response is sought from a greater breadth of creative resource including, but not limited to communities. In this way, the path forward comes into sharper focus through the amalgamation of the shadows of death and the light of those living and working within them.

New public health approaches provide a lens through which we can begin to view and understand death in relation to the whole. How we now progress knowledge in this area is of vital importance. Do we conform to the model of evidence-based practice, showcasing compassionate communities and compassionate cities as an intervention or an entity to be evaluated? Or do we see them as a lens through which people can view and evaluate their own actions helping to build trust, membership and ‘know how’ to develop community capacity in real terms.

Such work transcends not only the academic and professional disciplines, but also cultural and spiritual boundaries. Herein lies perhaps the most significant problem. In a field seeking to incorporate such diverse philosophies, it can be difficult to forge a unified, collective approach without generating tensions. In the quest for a solution to what can feel like an impending crisis of care, we must acknowledge such tensions, work with them and through them where possible. However, we must also not fail to accept that such tensions are indeed the essence of what gives life to a situation. In the words of the Sufi poet Rumi ‘Out beyond ideas of right and wrong, there is a field. I’ll meet you there’. While this review goes some distance in bringing to light the complexity and tensions inherent within new public health approaches, what is clear is that if we take a reductionist approach and ignore them in our quest for an idealized ‘perfect’ system we will soon return to the place from which we began.
